# Structural and Computationally Driven Molecule Design in Drug Discovery: Current Advances and Future Perspectives

**DOI:** 10.3390/ph19081132

**Published:** 2026-07-23

**Authors:** Belgin Sever, Hasan DeMirci, Halilibrahim Ciftci

**Affiliations:** 1 Department of Pharmaceutical Chemistry, Faculty of Pharmacy, Anadolu University, 26470 Eskisehir, Türkiye; 2 Department of Molecular Biology and Genetics, Burdur Mehmet Akif Ersoy University, 15200 Burdur, Türkiye; hciftci@mehmetakif.edu.tr; 3 Department of Molecular Biology and Genetics, Koc University, Istanbul 34450, Türkiye; hdemirci@ku.edu.tr; 4 Stanford PULSE Institute, SLAC National Laboratory, Menlo Park, CA 94025, USA; 5 Medicinal and Biological Chemistry Science Farm Joint Research Laboratory, Faculty of Life Sciences, Kumamoto University, Kumamoto 862-0976, Japan; 6 Department of Drug Discovery, Science Farm Ltd., Kumamoto 862-0976, Japan

## 1. Introduction

Over the past two decades, drug discovery has undergone a remarkable transformation, evolving from a predominantly empirical endeavor into an increasingly rational and multidisciplinary scientific enterprise. Advances in structural biology, computational chemistry, medicinal chemistry, and molecular pharmacology have fundamentally reshaped the way therapeutic candidates are identified, designed, and optimized. Rather than functioning as isolated disciplines, these approaches have become closely interconnected, enabling researchers to investigate molecular interactions at atomic resolution, predict ligand-target recognition, prioritize promising compounds, and refine lead molecules before extensive experimental evaluation [1,2,3,4,5].

Among these advances, structure-based drug design has become one of the principal driving forces of modern pharmaceutical research. The rapid development of high-resolution structural techniques, advanced molecular simulations, artificial intelligence (AI), and accurate protein structure prediction has substantially expanded the possibilities for rational therapeutic design, enabling researchers to investigate biomolecular interactions with unprecedented precision and efficiency [1,2,3,4,5,6,7,8].

This multidisciplinary philosophy is clearly reflected in the contributions collected in the Special Issue (SI) “Structural and Computationally Driven Molecule Design in Drug Discovery: 2nd Edition”. Although the published articles address diverse therapeutic areas, including cancer, infectious diseases, neurodegenerative disorders, antimicrobial resistance, and metabolic diseases, they share a common objective: the application of structural and computational methodologies to facilitate rational molecular design and improve the efficiency of drug discovery. Collectively, these studies illustrate how complementary computational and experimental approaches can provide mechanistic insight, accelerate lead optimization, and ultimately contribute to the identification of biologically active therapeutic candidates.

Rather than providing a comprehensive summary of every contribution, this Editorial highlights the major scientific themes emerging from the SI and discusses their significance within the broader landscape of contemporary pharmaceutical research. 

## 2. Highlights from the Special Issue

One of the dominant themes emerging from this SI is the continued importance of structure-guided medicinal chemistry in anticancer drug discovery. Protein kinases remain among the most attractive therapeutic targets owing to their central roles in tumor progression and therapeutic resistance. Within this context, Reymova et al. developed a series of novel pyrimidine-tethered derivatives targeting the epidermal growth factor receptor (EGFR), integrating computational modeling with chemical synthesis and biological evaluation to support rational lead optimization [9]. Similarly, Sever and Ciftci reported the design and biological evaluation of *N*,*N*-diphenylaniline-based derivatives as Abelson tyrosine kinase (ABL) inhibitors against chronic myeloid leukemia (CML), demonstrating how molecular modeling can effectively complement medicinal chemistry in identifying promising kinase inhibitors [10]. Although these studies focus on different molecular targets, both illustrate a common strategy in contemporary anticancer drug discovery in which computational analyses are integrated with experimental investigations throughout the optimization process rather than being used solely as predictive tools.

Beyond kinase-targeted anticancer research, the contributions collected in this SI demonstrate the remarkable versatility of computationally driven drug discovery across numerous therapeutic areas, including infectious diseases, antimicrobial resistance, neurodegenerative disorders, and metabolic diseases. Together, these studies illustrate the broad applicability of molecular docking, molecular dynamics simulations, pharmacophore modeling, virtual screening, and AI for identifying biologically active molecules and supporting rational lead optimization.

The articles published in this SI demonstrate that successful drug discovery increasingly depends on the effective integration of computational prediction with experimental validation. Rather than functioning as isolated techniques, computational methodologies have become indispensable components of multidisciplinary discovery pipelines connecting structural biology, medicinal chemistry, chemical synthesis, and biological evaluation.

Although the studies presented in this SI originate from different research groups and address diverse biological targets, they consistently reveal a common scientific philosophy. Computational analyses are no longer viewed as isolated endpoints but rather as integral components of iterative drug discovery workflows that connect structural biology, medicinal chemistry, chemical synthesis, and biological evaluation. 

## 3. Future Perspectives

The future of drug discovery will likely be defined not by the development of individual computational methodologies, but by their seamless integration with structural biology and experimental pharmacology. Recent advances in AI, next-generation protein structure prediction, and structure-guided molecular design are expected to accelerate target identification, lead optimization, and translational research while simultaneously reducing development time and cost [4,5,6,7,8].

Nevertheless, computational prediction alone is insufficient. The ultimate success of modern drug discovery will continue to depend on rigorous experimental validation capable of transforming computational hypotheses into clinically relevant therapeutic candidates.

From our perspective, one of the most encouraging observations during the preparation of this SI was the remarkable convergence of independent research groups toward multidisciplinary research strategies. Although the published studies investigated different diseases and molecular targets, they consistently integrated computational modeling, structural biology, medicinal chemistry, and biological evaluation. This convergence suggests that future pharmaceutical research will increasingly rely on collaborative scientific ecosystems capable of translating molecular insights into therapeutic innovation.

## 4. Conclusions

The studies collected in the SI “Structural and Computationally Driven Molecule Design in Drug Discovery: 2nd Edition” provide a timely overview of the current landscape of structure-guided and computationally driven pharmaceutical research. Although the contributions encompass diverse therapeutic indications and methodological approaches, they collectively emphasize a common scientific principle: meaningful advances in drug discovery arise through the effective integration of computational methodologies with structural biology, medicinal chemistry, and experimental validation.

The selected articles demonstrate that computational approaches are no longer limited to supporting molecular modeling or virtual screening but have become essential components of rational drug discovery workflows. Equally important, they highlight the continuing need for experimental confirmation to translate computational predictions into biologically and therapeutically relevant outcomes. 

We hope that the contributions presented in this SI will stimulate further interdisciplinary collaborations, encourage the development of innovative computational and structural methodologies, and ultimately contribute to the discovery of safer and more effective therapeutic agents. As Guest Editors, we sincerely thank all authors, reviewers, and the editorial team for their valuable contributions, dedication, and support, which made this SI possible.

## 5. List of Contributions

The Special Issue “Structural and Computationally Driven Molecule Design in Drug Discovery: 2nd Edition” includes the following contributions:

1. Alkhatabi, H.A.; Alatyb, H.N. Pharmacophore-Based Study: An In Silico Perspective for the Identification of Potential New Delhi Metallo-β-lactamase-1 (NDM-1) Inhibitors. *Pharmaceuticals* **2024**, *17*, 1183. https://doi.org/10.3390/ph17091183.
2. Shamsi, A.; Shahwan, M.; Zuberi, A.; Altwaijry, N. Identification of Potential Inhibitors of Histone Deacetylase 6 Through Virtual Screening and Molecular Dynamics Simulation Approach: Implications in Neurodegenerative Diseases. *Pharmaceuticals* **2024**, *17*, 1536. https://doi.org/10.3390/ph17111536.
3. Hassan, A.M.; Gattan, H.S.; Faizo, A.A.; Alruhaili, M.H.; Alharbi, A.S.; Bajrai, L.H.; Al-Zahrani, I.A.; Dwivedi, V.D.; Azhar, E.I. Evaluating the Binding Potential and Stability of Drug-like Compounds with the Monkeypox Virus VP39 Protein Using Molecular Dynamics Simulations and Free Energy Analysis. *Pharmaceuticals* **2024**, *17*, 1617. https://doi.org/10.3390/ph17121617
4. Marafie, S.K.; Alshawaf, E.; Al-Mulla, F.; Abubaker, J.; Mohammad, A. Targeting mTOR Kinase with Natural Compounds: Potent ATP-Competitive Inhibition Through Enhanced Binding Mechanisms. *Pharmaceuticals* **2024**, *17*, 1677. https://doi.org/10.3390/ph17121677.
5. Elsaman, T.; Mohamed, M.A. Examining Prenylated Xanthones as Potential Inhibitors Against Ketohexokinase C Isoform for the Treatment of Fructose-Driven Metabolic Disorders: An Integrated Computational Approach. *Pharmaceuticals* **2025**, *18*, 126. https://doi.org/10.3390/ph18010126.
6. Reymova, F.; Topalan, E.; Sever, B.; Sevimli-Gur, C.; Can, M.; Tuyun, A.F.; Başoğlu, F.; Ece, A.; Otsuka, M.; Fujita, M.; Demirci, H.; Ciftci, H. Design, Synthesis, and Mechanistic Anticancer Evaluation of New Pyrimidine-Tethered Compounds. *Pharmaceuticals* **2025**, *18*, 270. https://doi.org/10.3390/ph18020270.
7. Mohamed, M.S.; Elsaman, T.; Mohamed, M.A.; Eltayib, E.M.; Abdalla, A.E.; Idriss, M.T. Identification of Bacterial Oligopeptidase B Inhibitors from Microbial Natural Products: Molecular Insights, Docking Studies, MD Simulations, and ADMET Predictions. *Pharmaceuticals* **2025**, *18*, 709. https://doi.org/10.3390/ph18050709.
8. Hakeem, I.J.; Alahdal, H.; Baeissa, H.M.; Bakhsh, T.; Rafeeq, M.; Habib, A.H.; Karami, M.M.; Al-Ghamdi, M.A.; Abdullah, G.; Al Tuwaijri, A. Unveiling the Polypharmacological Potency of FDA-Approved Rebamipide for Alzheimer’s Disease. *Pharmaceuticals* **2025**, *18*, 772. https://doi.org/10.3390/ph18060772.
9. Alici, H. Structure-Based Design and In Silico Evaluation of Computationally Proposed Curcumin Derivatives as Potential Inhibitors of the Coronaviral PLpro Enzymes. *Pharmaceuticals* **2025**, *18*, 798. https://doi.org/10.3390/ph18060798.
10. Kawsar, S.M.A.; Al-Mijalli, S.H.; Bouzid, G.; Abdallah, E.M.; Siddiquee, N.H.; Hosen, M.A.; Horchani, M.; Ghalla, H.; Jannet, H.B.; Fujii, Y.; Ozeki, Y. Unveiling Palmitoyl Thymidine Derivatives as Antimicrobial/Antiviral Inhibitors: Synthesis, Molecular Docking, Dynamic Simulations, ADMET, and Assessment of Protein–Ligand Interactions. *Pharmaceuticals* **2025**, *18*, 806. https://doi.org/10.3390/ph18060806.
11. Alici, H.; Topuz, S.; Demir, K.; Taslimi, P.; Tahtaci, H. Synthesis, Biological Evaluation, and In Silico Characterization of Novel Imidazothiadiazole–Chalcone Hybrids as Multi-Target Enzyme Inhibitors. *Pharmaceuticals* **2025**, *18*, 962. https://doi.org/10.3390/ph18070962.
12. Alghamdi, M.A. From Molecules to Medicines: The Role of AI-Driven Drug Discovery Against Alzheimer’s Disease and Other Neurological Disorders. *Pharmaceuticals* **2025**, *18*, 1041. https://doi.org/10.3390/ph18071041.
13. Abdelrahman, A.H.M.; Mekhemer, G.A.H.; Sidhom, P.A.; Abalkhail, T.; Khan, S.; Ibrahim, M.A.A. In Silico Mining of the Streptome Database for Hunting Putative Candidates to Allosterically Inhibit the Dengue Virus (Serotype 2) RdRp. *Pharmaceuticals* **2025**, *18*, 1135. https://doi.org/10.3390/ph18081135.
14. Sever, B.; Ciftci, H.I. Design, Synthesis, and Biological Evaluation of *N*,*N*-Diphenylaniline-Based Derivatives as Antiproliferative Agents and ABL TK Inhibitors Against CML. *Pharmaceuticals* **2026**, *19*, 416. https://doi.org/10.3390/ph19030416.

## Data Availability

No new data were created or analyzed in this study. Data sharing is not applicable to this article.
